# Microsurgical Outcomes in 1000 Patients With Cerebellopontine Angle Tumors: A Comprehensive Cohort Analysis

**DOI:** 10.1002/ohn.70016

**Published:** 2025-09-08

**Authors:** Joshua Lee, Michael G. Brandel, Benjamin T. Ostrander, Philipp Verpukhovskiy, Alena Pauley, Abhishek Bhatt, Alexandra Vacaru, Douglas M. Bennion, Marc S. Schwartz, Rick Friedman

**Affiliations:** ^1^ Department of Otolaryngology–Head and Neck Surgery University of California, San Diego La Jolla California USA; ^2^ Department of Otolaryngology–Head and Neck Surgery University of Iowa Iowa City Iowa USA; ^3^ Department of Neurosurgery University of California, San Diego La Jolla California USA

**Keywords:** acoustic neuroma, cerebellopontine angle tumors, microsurgery outcomes, vestibular schwannoma

## Abstract

**Objective:**

To summarize the outcomes of 1000 consecutive microsurgical resection of cerebellopontine angle tumors.

**Study Design:**

Retrospective cohort study.

**Setting:**

Single tertiary care institution.

**Methods:**

We analyzed 1000 patients who underwent microsurgical resection of cerebellopontine angle tumors between November 2017 and August 2024. Patient and tumor‐related characteristics are summarized, and the extent of resection, facial nerve function, hearing preservation, and postoperative complications are described. Volumetric analysis was used to assess resection completeness.

**Results:**

The median patient age was 49 years (interquartile range [IQR] 40‐58), with 62% female and 74% Caucasian. Surgical approaches included 46.5% translabyrinthine (TL), 24.6% retrosigmoid (RS), 24.1% middle cranial fossa (MCF), and 4.8% other. The median tumor size was 19 mm (IQR 12‐27), and 52% were left‐sided. Overall, volumetric analysis for vestibular schwannoma tumors revealed that 88% of patients had ≤2% tumor volume remaining, 4% had >2% and ≤5%, 4% had >5% and ≤10%, and 4% had >10%. Additionally, the average extent of resection was 98% volume reduction. The most common postoperative complication was cerebrospinal fluid leak (10%), with 77% of cases treated by lumbar drain. Postoperative House‐Brackmann (HB) scores of I or II were achieved in 88% of patients, which had improved to 90% at the time of last follow‐up. Hearing preservation was 62% for MCF and 33% for RS cases.

**Conclusion:**

Microsurgical resection of cerebellopontine angle tumors yields favorable outcomes, particularly in preserving facial nerve function and hearing. Tumor size and surgical approach impacted hearing preservation, with the middle fossa approach being most effective for smaller tumors.

Cerebellopontine angle (CPA) tumors, including vestibular schwannomas (VSs) and meningiomas, are largely benign extra‐axial neoplasms that emerge in the anatomically sacrosanct region between the cerebellum and the pons. Patients often present with hearing loss, tinnitus, disequilibrium, and vertigo episodes.^1^ VSs comprise 75% to 85% of all CPA tumors.^2^, ^3^, ^4^ VSs are slow‐growing tumors that arise from the vestibulocochlear nerve due to an over‐proliferation of local Schwann cells. Current incidence estimates range from 1.1 to 1.7 cases per 100,000 people, which has increased in recent decades, thought to be due largely to advances in magnetic resonance imaging (MRI) and incidental identification of small intracanalicular tumors.^5^, ^6^ This epidemiologic shift underscores the importance of identifying and optimizing the most effective treatment strategies for CPA tumors at all stages from small and asymptomatic to large and disabling.^7^, ^8^, ^9^

Management options for CPA tumors include observation, radiosurgery, or microsurgery, and treatment decisions are influenced by many factors, including tumor size and location, patient age, patient preference, surgeon experience, and preoperative hearing status.^10^, ^11^ A “watch and wait” approach, involving surveillance with neuroimaging every 6 to 12 months, is generally indicated when a patient presents with minimal symptoms and small tumors, although early surgical removal results in improved hearing preservation rates among appropriately selected patients.^12^ In contrast, tumors that are growing or associated with moderate or severe symptoms typically warrant timely intervention: radiosurgery versus microsurgical resection.^13^, ^14^ More recently, treatment strategies have prioritized cranial nerve (CN) function over extent of resection (EOR), including increased selection of primary stereotactic radiosurgery or planned subtotal resection (STR).^7^, ^13^, ^15^, ^16^ These strategies are more likely to require adjuvant treatment or revision surgery, which is associated with substantially increased risk of complications, including facial nerve weakness.^17^, ^18^ In studies of radiosurgery for VS, the primary goal of controlled tumor growth is achieved in more than 95% of patients, with adverse effects ranging by study and including facial nerve deficits (0%‐21%), trigeminal nerve dysfunction (0.3%‐27%), and hearing loss (75%‐80%) when assessed 3 to 5 years after treatment.^19^

This study aims to contribute to the ongoing discussion of optimal management strategies by reporting our series of 1000 CPA tumors of all sizes treated by microsurgical resection. We place particular emphasis on outcomes, including the extent of tumor resection, facial nerve function, hearing preservation, and complications.

## Methods

### Study Design and Patient Population

This study is a retrospective analysis conducted at a single tertiary care center (IRB#180978). Patients diagnosed with sporadic CPA tumors and treated by a single neurosurgeon/neurotologist team were enrolled between November 2017 and August 2024. As many patients come for consultation from other geographic regions, annual follow‐ups were typically conducted by phone with imaging review. To provide a comparison group, we reviewed the charts in alphabetical order of our tumor patients (ICD code D33.3) with San Diego zip codes since 2017 to identify 100 CPA tumor patients for whom nonoperative management was recommended. Patients with tumors not involving the CPA or with neurofibromatosis type 2 (NF2) were excluded. Demographic and tumor‐related data were extracted from electronic medical records. Tumor dimensions and hearing levels were obtained from preoperative MRIs and audiograms.

### Surgical Outcomes

EOR was calculated by volumetric analysis of preoperative and postoperative MRIs for all patients who underwent non‐gross total resection (GTR), as determined clinically. Facial nerve function using the House‐Brackmann (HB) grading system was recorded immediately postoperatively, at hospital discharge, at initial follow‐up, and at last follow‐up when available. Postoperative complications were documented through progress notes and patient follow‐ups. Assessment of long‐term clinical outcomes for many patients was limited by proximity to the surgery date or due to telemedicine follow‐up limitations.

### Hearing Preservation

Hearing preservation was defined as a postoperative four‐frequency pure tone average (500, 1000, 2000, and 3000 Hz) of ≤50 dB HL and a word recognition score of ≥50%, corresponding to class A and B hearing from the AAO‐HNS hearing classification guidelines or a postoperative word recognition score reduction <10% from baseline.^20^, ^21^

### Volumetric Analysis

For clinically assessed GTR, 100% resection was assumed. Among 894 VS patients, 237 underwent non‐GTR with 84 patients excluded due to missing follow‐up scans. For the remaining non‐GTR patients, post‐contrast T1‐weighted MRIs with the thinnest available slice thickness (typically 0.5 mm) were used to perform tumor segmentation and volume calculations with Visage 3D segmentation version 7.1.19.^22^ Two independent reviewers performed segmentation of preoperative scans. If segmentation results differed by <10%, the values were averaged. For discrepancies >10%, segmentation was adjudicated by a third reviewer. Postoperative segmentations were also performed. EOR was calculated as:

$$\frac{\mathrm{pre}‐\mathrm{op}\;\mathrm{volume}-\mathrm{post}‐\mathrm{op}\;\mathrm{volume}}{\mathrm{pre}‐\mathrm{op}\;\mathrm{volume}}.$$

### Statistical Analysis and Sample Size Calculation

Descriptive statistics were used to summarize demographics, tumor characteristics, and outcomes.

## Results

### Patient Demographics

In total, 1000 surgical patients diagnosed with sporadic CPA tumors were included (Table 1). Median age at surgery was 48.7 years (interquartile range [IQR] 39.5‐58.3 years); 62% were female, 74% were Caucasian, and 91% identified as non‐Hispanic. For comparison, 100 nonsurgical patients were also included (Table 2). Median age of these patients at 66.7 years was significantly older, and tumors were of a significantly smaller maximum dimension (10.1 vs 19.3 mm) than for patients managed with surgery (*P* < .0001). Among these patients, 12% were recommended for radiation treatment, whereas the remainder were managed with observation. Given these significant between‐group differences and the intent to focus on surgical outcomes, further analyses were performed among patients from the surgical group.

**Table 1:** Summary of Surgical Patient‐ and Tumor‐Related Characteristics

	All patients (n = 1000)	Translabyrinthine resection (n = 465)	Retrosigmoid resection (n = 246)	Middle fossa resection (n = 241)	Other resection (n = 48)
Age, median years (IQR)	48.7 (39.5, 58.3)	50.4 (38.7, 61.5)	48.9 (40.4, 56.4)	47.2 (39.8, 47.2)	44.6 (36.2, 54.9)
Weight, median lbs (IQR)	166.4 (140.5, 197.75)	168.5 (140, 198.1)	165 (12.25, 198.75)	172 (144, 198.9)	148 (135.83, 172.93)
Height, median inches (IQR)	67 (64, 70)	66 (64, 70)	67 (65, 71)	66.25 (64, 69.25)	66 (63, 68.13)
Sex, n (%)					
Female	620 (62%)	281 (60.4%)	148 (60.2%)	153 (63.5%)	40 (83.3%)
Male	380 (38%)	184 (39.6%)	98 (39.8%)	88 (36.5%)	8 (16.7%)
Race, n (%)					
American Indian/Alaska Native	2 (0.2%)	1 (0.2%)	0 (0%)	0 (0%)	1 (2.1%)
Asian	83 (8.3%)	43 (9.2%)	22 (8.9%)	13 (5.4%)	5 (10.4%)
Black or African American	13 (1.3%)	6 (1.3%)	2 (0.8%)	4 (1.7%)	1 (2.1%)
Native Hawaiian/Pacific Islander	3 (0.3%)	1 (0.2%)	0 (0%)	2 (0.8%)	0 (0%)
Other race or mixed race	116 (11.6%)	53 (11.4%)	26 (10.6%)	31 (12.9%)	6 (12.5%)
Unknown (patient refuses to declare race)	43 (4.3%)	17 (3.7%)	12 (4.9%)	12 (5%)	2 (4.2%)
Caucasian	740 (74%)	344 (74%)	184 (74.8%)	179 (74.3%)	33 (68.8%)
Hispanic or non‐Hispanic, n (%)					
Non‐Hispanic	894 (89.4%)	413 (88.8%)	223 (90.7%)	221 (91.7%)	37 (77.1%)
Hispanic	86 (8.6%)	45 (9.7%)	14 (5.7%)	17 (7.1%)	10 (20.8%)
Did not report	20 (2%)	7 (1.5%)	9 (3.6%)	3 (1.2%)	1 (2.1%)
Smoking status, n (%)					
Current every day smoker	13 (1.7%)	6 (1.7%)	4 (2.1%)	3 (1.6%)	0 (0%)
Current some day smoker	12 (1.6%)	6 (1.7%)	2 (1%)	2 (1%)	2 (8.3%)
Former smoker	120 (15.6%)	61 (17%)	27 (13.9%)	29 (15%)	3 (12.5%)
Light tobacco smoker	1 (0.1%)	1 (0.3%)	0 (0%)	0 (0%)	0 (0%)
Never smoker	615 (79.9%)	282 (78.6%)	159 (82%)	155 (80.3%)	19 (79.2%)
Never assessed	9 (1.2%)	3 (0.8%)	2 (1%)	4 (2.1%)	0 (0%)
History of prior surgery, n (%)					
Yes	57 (5.7%)	43 (9.2%)	3 (1.2%)	5 (2.1%)	6 (12.5%)
No	943 (94.3%)	422 (90.8%)	243 (98.8%)	236 (97.9%)	42 (87.5%)
History of prior radiation therapy, n (%)					
Yes	51 (5.1%)	44 (9.5%)	2 (0.8%)	2 (0.8%)	3 (6.3%)
No	949 (94.9%)	421 (90.5%)	244 (99.2%)	239 (99.2%)	45 (93.8%)
CN VII integrity (%)					
Intact	98.1%	96.3%	100%	100%	95.8%
Not intact	1.9%	3.7%	0%	0%	4.2%
CN VIII integrity (%)					
Intact	89.7%	‐	83.1%	96%	‐
Not intact	10.3%	‐	16.9%	4%	‐
Postoperative complications, n (%)					
Cerebrospinal fluid (CSF) leak	100 (10%)	50 (10.8%)	21 (8.5%)	24 (10%)	5 (10.4%)
Requiring lumbar drain	77 (77% of CSF leak patients)	36 (72% of CSF leak patients)	17 (81% of CSF leak patients)	19 (79% of CSF leak patients)	5 (100% of CSF leak patients)
Intracranial hemorrhage (bleed)	2 (0.2%)	2 (0.4%)	0 (0%)	0 (0%)	0 (0%)
Edema	28 (2.8%)	20 (4.3%)	8 (3.3%)	0 (0%)	0 (0%)
Ischemic CVA	4 (0.4%)	3 (0.6%)	1 (0.4%)	0 (0%)	0 (0%)
Infection/meningitis	11 (1.1%)	6 (12.9%)	2 (0.8%)	3 (1.2%)	0 (0%)
Dural sinus thrombosis	7 (0.7%)	7 (1.5%)	0 (0%)	0 (0%)	0 (0%)
Postoperative seizure	1 (0.1%)	1 (0.2%)	0 (0%)	0 (0%)	0 (0%)
Tumor dimensions, median (IQR)	19 (12, 27)	23 (14, 30)	18 (14, 22)	9 (6, 12)	32 (20.5, 38.5)
Tumor laterality, n (%)					
Left	523 (52.3%)	235 (50.5%)	133 (54.1%)	127 (52.7%)	28 (58.3%)
Right	477 (47.7%)	230 (49.5%)	113 (45.9%)	114 (47.3%)	20 (41.7%)
Pathology, n (%)					
Vestibular schwannoma	894 (89.4%)	435 (93.5%)	232 (94.3%)	221 (91.7%)	6 (12.5%)
Meningioma WHO grade I	57 (5.7%)	12 (2.6%)	5 (2%)	12 (5%)	28 (58.3%)
Other	49 (4.9%)	18 (3.9%)	9 (3.7%)	8 (3.3%)	14 (29.2%)
Facial nerve outcomes (1‐2 wk post‐op visit), n (%)					
HB I	723 (74.5%)	303 (69.5%)	201 (81.7%)	187 (77.6%)	32 (68.1%)
HB II	113 (11.7%)	59 (13.5%)	25 (10.2%)	24 (10%)	5 (10.6%)
HB III	34 (3.5%)	18 (4.1%)	4 (1.6%)	9 (3.7%)	3 (6.4%)
HB IV	32 (3.3%)	19 (4.4%)	4 (1.6%)	9 (3.7%)	0 (0%)
HB V	25 (2.6%)	13 (3.0%)	4 (1.6%)	6 (2.5%)	2 (4.3%)
HB VI	43 (4.4%)	24 (5.5%)	8 (3.3%)	6 (2.5%)	5 (10.6%)
Hearing preservation, n (%)					
Preserved	221 (48%)	‐	77 (33%)	144 (62%)	‐
Not preserved	243 (52%)	‐	154 (67%)	89 (38%)	‐

Abbreviations: CN, cranial nerve; CVA, cerebrovascular accident; HB, House‐Brackmann; IQR, interquartile range; WHO, World Health Organization.

**Table 2:** Summary of Nonsurgical Patient‐ and Tumor‐Related Characteristics

	Surgical patients (n = 1000)	Nonsurgical patients (n = 100)	*P* value
Age, median years (IQR)	48.7 (39.5, 58.3)	69 (60, 76)	*P* < .0001
Sex, n (%)			
Female	620 (62%)	52 (52%)	*P* < .0001
Male	380 (38%)	48 (48%)	*P* < .0001
Tumor dimensions, median mm (IQR)	19 (12, 27)	9 (4, 15)	*P* < .0001

Abbreviation: IQR, interquartile range.

### Surgical Approach and Outcomes

Surgical approaches included translabyrinthine (TL; 46.5%), retrosigmoid (RS; 24.6%), middle fossa (24.1%), and other (4.8%). EOR determined by clinical assessment is displayed in Supplemental Table S1, available online.

CN VIII preservation was achieved in 90% of middle fossa and RS approach cases. CN VII integrity was maintained in 98% of cases. In patients with non‐intact CN VII, the mean tumor size was 28.7 mm, with 42% having prior surgery or radiation.

### EOR and Volumetric Analysis

Volumetric analysis for non‐GTR VS revealed that 88% of patients had ≤2% tumor volume remaining, 4% had >2% and ≤5%, 4% had >5% and ≤10%, and 4% had >10%. The average EOR for these tumors was 98% (Table 3). EOR based on clinical definitions and volumetric analysis is demonstrated in Supplemental Figure S1, available online.

**Table 3:** Extent of Resection by Percentage of Tumor Volume Remaining for All Vestibular Schwannoma Patients Only Using Volumetric Analysis With Further Stratification by Surgical Approach

Tumor volume remaining	All VS patients	Translabyrinthine	Retrosigmoid	Middle fossa	Other
≤2% volume remaining	716 (88.3%)	301 (80.3%)	197 (91.2%)	215 (99.1%)	3 (100%)
>2% and ≤5% volume remaining	36 (4.4%)	29 (7.7%)	7 (3.2%)	0	0
>5% and ≤10% volume remaining	27 (3.3%)	23 (6.1%)	4 (1.9%)	0	0
>10% volume remaining	32 (4.0%)	22 (5.9%)	8 (3.7%)	2 (0.9%)	0

Abbreviation: VS, vestibular schwannoma.

### Postoperative Complications

The most common postoperative complications, shown in Table 1, included cerebrospinal fluid (CSF) leak (10%) and infection/meningitis (1%). Of those with CSF leaks, 77% required the placement of a lumbar drain. There were no mortalities.

### Tumor Characteristics

The median tumor dimension was 19 mm (IQR 12‐27 mm), with approach‐specific medians of 23 mm (TL), 18 mm (RS), and 9 mm (middle fossa). Prior surgery and radiation were reported in 6% and 5% of cases, respectively. Pathology identified 89% as VS, 6% as World Health Organization (WHO) grade I meningiomas, and 5% as other types (Table 4).

**Table 4:** Tumor Type Based on Pathology Report Shown Including Vestibular Schwannoma, Meningioma World Health Organization (WHO) Grade I, and Other Tumor Types Along With Their Respective Counts

Tumor pathology type	Count (n)
Vestibular schwannoma	894
Meningioma WHO grade I	57
Other	49
Arachnoid cyst	1
Benign fibro‐osseous lesion	1
Benign peripheral nerve with focal calcification and ganglion	1
Cavernoma	1
Cavernous hemangioma	1
Clear cell meningioma	1
Endolymphatic sac papillary tumor	1
Epidermoid cyst	1
Facial schwannoma	6
Ganglioneuroma	1
Granulomatous and necrotizing vasculitis	1
Hemangioblastoma	2
Hemangioma	1
Jugular foramen schwannoma	3
Large grade 2 atypical choroid plexus papilloma	1
Left 8th nerve lipoma	1
Left cerebellopontine angle tumor (low‐grade myxomatous spindle cell neoplasm)	1
Lipochoristoma	1
Lipoma	1
Low‐grade chondrosarcoma	1
Lower cranial nerve schwannoma	1
Lower cranial schwannoma	1
Malignant peripheral nerve sheath tumor	1
Malignant peripheral nerve sheath tumor, high‐grade	1
Meningioma WHO grade II	6
Meningioma WHO grade III	2
MVD of glossopharyngeal nerve	2
Rosai‐Dorfman disease	1
Sarcoma	1
Transitional subtype meningioma	1
Trigeminal schwannoma	4

Abbreviation: MVD, microvascular decompression.

### Facial Nerve Outcomes

Facial nerve function, assessed by the HB grading system, showed that 97% of patients had an HB grade I or II preoperatively. Any patients with preexisting HB grade of VI were excluded from the analysis of facial nerve outcome. At the time of discharge after surgery, 86% of patients had retained HB grade I or II. Among 391 patients with long‐term follow‐up, 87% had HB I/II.

Among 162 patients with postoperative HB grades III to VI, 16.7% (27/162) had preexisting facial nerve weakness. Of these, 1 patient had improvement, and 17 maintained baseline function. Additionally, 20% (32/162) of patients with postoperative HB grades III to VI showed improvement to HB grades I or II on long‐term follow‐up, none of whom had preexisting facial nerve weakness. If all patients with preoperative facial nerve weakness are excluded, the proportion of postoperative HB I/II increased to 90% (872/973).

Facial nerve outcomes were analyzed by surgical approach. Patients who underwent the RS approach demonstrated HB I/II in 92%. Among middle cranial fossa (MCF) patients, HB I/II was seen in 88%. For the TL approach, HB I/II was seen in 78%. Median tumor size for patients with postoperative HB I/II was 16 mm, compared to 22 mm for those with HB III to VI.

### Hearing Preservation

In the MCF cohort, initial evaluation of hearing preservation based solely on the preoperative versus early postoperative period showed that 139/241 patients retained functional hearing, which increased to 144/241 on delayed follow‐up. Excluding three patients who did not undergo postoperative audiograms and accounting for five patients who had surgery but without tumor resection, the overall hearing preservation rate was 62%.

For patients who underwent the RS approach for larger tumors, hearing preservation was achieved in 73/246 patients initially, which later increased to 77/246. Following the exclusion of 11 patients who lacked postoperative audiograms and 4 patients who had surgery without tumor resection, the final hearing preservation rate was 33%.

Hearing preservation varied based on tumor size and surgical approach. Among MCF patients, hearing preservation rates were highest for those with tumors ≤5 mm at 75%, followed by 66% for tumors between 5.1 and 10 mm, and 51% for tumors larger than 10 mm. For patients with superior vestibular nerve tumors, the hearing preservation rate was 83%.^23^ For patients treated via the RS approach, hearing preservation was 43% for tumors ≤15 mm, 29% for tumors 15.1 to 20 mm, and 29% for those >20 mm.

Hearing preservation also varied by tumor type. Among patients who underwent resection of non‐VS tumors, 29 of 36 (81%) with both preoperative and postoperative audiograms demonstrated immediate hearing preservation. In contrast, for patients who underwent resection of VS tumors via either the MCF or RS approach, 212 of 464 (46%) had preserved hearing postoperatively. This difference was statistically significant (*P* < .0005), suggesting that patients with non‐VS tumors were more likely to retain hearing after surgery than those with VS tumors.

Of note, in both the MCF and RS groups, the few cases in which no tumor resection was performed were due to alternate diagnoses that did not warrant resection, such as facial schwannoma, lipochoristoma, or benign peripheral nerve lesions with focal calcification.

## Discussion

This retrospective case series presents outcomes in 1000 consecutive patients undergoing microsurgery for sporadic CPA tumors, with results demonstrating low complication rates and favorable clinical outcomes, including high rates of gross or near‐total resection (NTR), good facial nerve function, and hearing preservation.

Decision‐making for VS management is highly individualized, recognizing that while some cases present clear treatment pathways, others require careful consideration of multiple factors. Tumor size, growth rate, symptom burden, patient age, overall health, and personal preferences influence recommendation for observation, microsurgical resection, or stereotactic radiation. The decision in many cases falls into a gray area where clinical equipoise exists. In these instances, shared decision‐making becomes critical, with discussions structured around whether an option is “recommended,” “reasonable,” or “possible but not recommended,” ensuring that patients have realistic expectations and feel confident in their choice.^19^

Comparison between groups of our surgically and nonsurgically managed patients reinforces the notion that patient age and tumor size are essential considerations in recommending appropriate management. Our analysis demonstrated that patients who did not undergo surgery were significantly older and had substantially smaller tumors (Table 2, *P* < .0001). At our center, nonsurgical patients are relatively fewer than surgical patients, as many surgery‐oriented patients electively come from elsewhere for consultation. Hence, the nonsurgical group was necessarily selected from San Diego‐area patients whose course of diagnosis, referral, and initial consultation was more typical. Given fundamental group differences in tumor size, demographics, and referral patterns, more extensive between‐group comparisons would be influenced by significant selection bias and confounding factors, limiting the validity of conclusions about operative versus nonoperative management.

Recent VS management has trended towards more conservative approaches. A retrospective analysis of the SEER database revealed that microsurgery has been used less frequently over time, observation was used more frequently, and the pattern of radiation therapy remained unchanged.^7^ The optimal treatment approach for maximizing quality of life (QOL) and preserving hearing remains a point of ongoing study, and four recent published studies on QOL after MCF resection were overwhelmingly positive.^24^

Numerous large case series and systematic reviews of VS treatment have been previously published. Ansari et al conducted a systematic review of 5064 patients receiving surgical treatment for VS, demonstrating that the MCF approach was superior to the RS approach for hearing preservation in patients with tumors < 1.5 cm (hearing loss in 43.6% vs 64.3%, *P* < .001) and the RS approach was associated with significantly less facial nerve dysfunction in patients with intracanalicular tumors than the MCF method (4% vs 16.7%, respectively, *P* < .001).^25^ In a retrospective review of 1167 large (>20 × 30 mm) VS treated through RS craniotomy, GTR was achieved in 1006 patients (86.2%), anatomical facial nerve preservation occurred in 1083 cases (92.8%), and functional facial nerve preservation by postoperative HB scale showed 423 patients (36.2%) in grades I to II, 534 cases (45.8%) in grade III, and 210 patients (18.0%) in grades IV to VI.^26^ In a case series published in 2016, Zhang et al reported their 17‐year experience with 1006 cases, with good facial nerve function at one year post‐op observed in 85.1% of patients (HB grades I‐II) and hearing preservation in 61.6% of patients. However, with follow‐up, useful hearing (classes A + B) was observed in 33.5% of cases overall.^27^

Matthies published a review of 1000 cases treated surgically in 1997, focusing on the evolution of clinical signs and symptoms, with the most common complications including CSF leak and headache more so in patients undergoing an RS approach.^28^ Our CSF leak rate of 10% aligns with findings from large studies, which also report similar usage of conservative management strategies, such as lumbar drains.^29^, ^30^, ^31^

In our series, facial nerve function, as assessed by the HB grading system, showed that 88% of patients had an HB grade I or II on postoperative day 1. If patients with preoperative facial nerve weakness are excluded, the postoperative HB scores improved to 90% (872/973) achieving HB grades I or II. Hearing preservation remains another major treatment goal in the treatment of VS. Our results revealed that hearing preservation outcomes varied based on tumor size and surgical approach (Table 1). Hearing preservation rates were highest for those with small tumors who underwent the middle fossa approach (75%). In a recent 2024 meta‐analysis examining the RS versus middle fossa approach for hearing and facial nerve preservation by Palavani et al, among 7228 patients, hearing preservation for intracanalicular tumors was 62% for the middle fossa and 46% for the RS approach. However, in small tumors (<1.5 cm), the middle fossa showed 59% hearing preservation, contrasting to 85% RS.^32^

The most influential factors affecting hearing preservation rates are tumor size and preoperative hearing status. Wallerius et al reported in a review of 243 patients treated with microsurgical resection, the strongest predictor of hearing preservation with microsurgery after multivariable adjustment is tumor size, with only 10% of patients with tumors ≥15 mm of CPA extension retaining serviceable hearing after microsurgery.^33^ In contrast, Ahmed et al examined the immediate postoperative and long‐term hearing outcome data in 155 patients who had undergone hearing preservation attempts with the MCF approach, among whom class A or B hearing was preserved in 70%. Further, in a subset of 71 patients monitored over a longer follow‐up period, class A or B hearing preservation was 82% at 3 to 5 years and declined thereafter, and word recognition score class I or II hearing preservation was 98% at 3 to 5 years and declined less rapidly thereafter.^34^ By way of comparison with other management strategies, one study examined long‐term hearing outcomes following stereotactic radiosurgery and found that estimated rates of serviceable hearing declined over time, falling from 80% at 1 year to 23% at 10 years.^35^ The rate of hearing preservation for observation over time was slightly better, with Kaplan‐Meier estimated rates of maintaining serviceable hearing at 1 and 10 years following diagnosis of 94% and 44%, respectively.^36^

### Volumetric Analysis

Subjective categorization of tumor resection volume by the operative surgeon is hampered by a lack of standardized definitions and the inherent variability in any one individual's ability to accurately estimate precise volumetric tumor reductions (see Supplemental Figure S1, available online). In a study by Jacob et al, the extent of tumor resection was defined objectively by volumetric analysis, with NTR assigned when a small tumor remnant no larger than 5 × 5 × 2 mm was intentionally left in an effort to preserve neural integrity.^37^ They found that the STR group had higher rates of tumor recurrence and growth compared to the NTR group. In our cohort, the average EOR for these tumors, as determined by volumetric analysis, was 98%, which may predict favorable rates of tumor recurrence/regrowth. The use of volumetric analyses of tumor reduction in this and future studies will allow for more accurate and objective quantification and comparison of tumor size and EOR.

### Limitations

This review reports outcomes from a single high‐volume center and surgical team. Extensive comparative analyses between our nonsurgical and surgical cohorts were not performed due to significant baseline differences in age, tumor size, and referral patterns, as our surgical cohort consisted of patients traveling nationally and internationally for operative intervention, whereas the nonsurgical cohort was composed of local patients of older ages and with smaller tumors, resulting in higher rates of conservative management. Surgeon bias regarding surgical approach is another limitation, which may skew results in favor of the surgeons’ preferred approach (eg, our team prefers TL as opposed to RS approach for large tumors). As a retrospective study, it is limited by missing data, specifically in long‐term facial nerve, hearing, and recurrence outcomes. Follow‐up studies are anticipated to help address these deficiencies though the broad geographic catchment of our center will impact these assessments.

## Conclusion

Here, we report on 1000 patients with sporadic CPA tumors treated with microvascular tumor removal. For patients with smaller intracanalicular tumors desiring hearing preservation, the middle fossa approach is safe and effective in our hands, offering the highest probability of preserved hearing (62%). For larger tumors with some CPA extension, the RS approach also offers a reasonable chance of hearing preservation (33%). All surgical approaches to the CPA have resulted in excellent rates of GTR and very low levels of complications, including permanent injury to the facial nerve and CSF leak.

## Author Contributions

**Joshua Lee**, investigation, data curation, formal analysis, writing—original draft, writing—review and editing, visualization, project administration. **Michael G. Brandel**, conceptualization, methodology, investigation, software, validation, formal analysis, writing—original draft, writing—review and editing, project administration. **Benjamin T. Ostrander**, Investigation, data curation, writing—original draft, writing—review and editing. **Philipp Verpukhovskiy**, investigation, writing—review and editing. **Alena Pauley**, investigation, writing—review and editing. **Abhishek Bhatt**, investigation, writing—review and editing. **Alexandra Vacaru**, investigation, writing—review and editing. **Douglas M. Bennion**, conceptualization, methodology, investigation, writing—original draft, writing—review and editing, visualization, supervision, project administration. **Marc S. Schwartz**, conceptualization, writing—review and editing, supervision. **Rick Friedman**, conceptualization, writing—review and editing, supervision.

## Disclosures

### Competing interests

The authors declare no conflicts of interest.

### Funding source

None.

## Supporting information

Supporting Information.

Supplemental Figure 1. Extent of VS resection by initial intraoperative clinical categorization (blue) versus volumetric analysis (orange). Gross total = 100%, near‐total = 95.1% to 99.9%, subtotal = 50.1% to 95%, partial = 0.1% to 50%, and no resection = 0%.
