# Supplementary material for: Microsurgical Outcomes in 1000 Patients With Cerebellopontine Angle Tumors: A Comprehensive Cohort Analysis
Source: Otolaryngol Head Neck Surg. 2025 Sep 8;173(5):1236–44. doi: 10.1002/ohn.70016 (PMC12574638; doi:10.1002/ohn.70016)
Supplement: Supplementary file 1 — Supporting Information. [file OHN-173-1236-s001.docx]

**Supplemental Table**

| **Extent of Resection, n (%)** | **All Patients** | **Translabyrinthine Resection** | **Retrosigmoid Resection** | **Middle Fossa Resection** | **Other Resection** | |
| --- | --- | --- | --- | --- | --- | --- |
| Gross Total | 723 (72.9%) | 272 (58.9%) | 198 (80.8%) | 224 (93.3%) | 29 (64.4%) |  |
| Near-Total | 21 (2.1%) | 8 (1.7%) | 6 (2.4%) | 3 (1.3%) | 4 (8.9%) |  |
| Subtotal | 207 (20.9%) | 160 (34.6%) | 33 (13.5%) | 7 (2.9%) | 7 (15.6%) |  |
| Partial | 29 (2.9% | 20 (4.3%) | 4 (1.6%) | 1 (0.4%) | 4 (8.9%) |  |
| None | 12 (1.2%) | 2 (0.4%) | 4 (1.6%) | 5 (2.1%) | 1 (2.2%) |  |

**Supplemental Table 1:** Extent of resection by intraoperative clinical assessment for all patients. Gross total = 100% resection, near-total = 95.1-99.9% resection, subtotal = 50.1-95% resection, partial = 0.1-50% resection, and no resection = 0% resection.
