# Supplementary figures and images for: Microsurgical Outcomes in 1000 Patients With Cerebellopontine Angle Tumors: A Comprehensive Cohort Analysis
Source: Otolaryngol Head Neck Surg. 2025 Sep 8;173(5):1236–44. doi: 10.1002/ohn.70016 (PMC12574638; doi:10.1002/ohn.70016)

## Extent of Resection

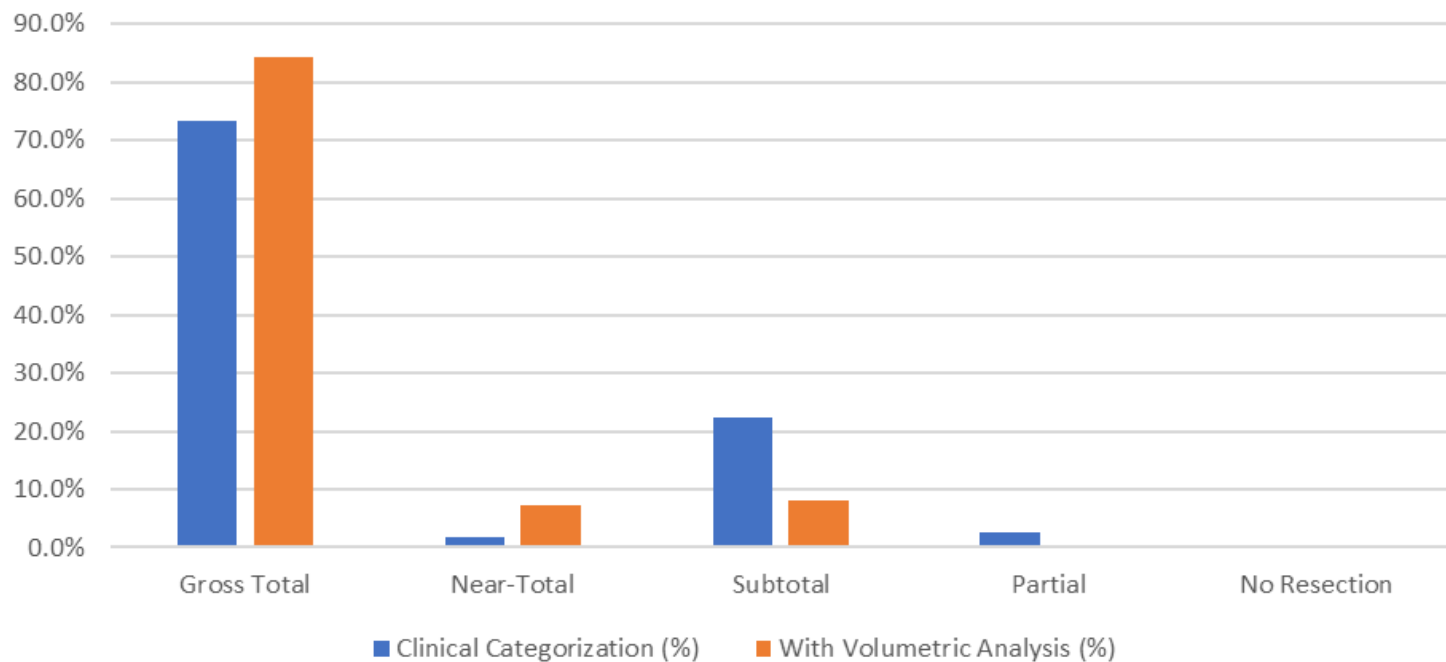

Supplement: Supplementary file 2 — Supplemental Figure 1. Extent of VS resection by initial intraoperative clinical categorization (blue) versus volumetric analysis (orange). Gross total = 100%, near‐total = 95.1% to 99.9%, subtotal = 50.1% to 95%, partial = 0.1% to 50%, and no resection = 0%. [file OHN-173-1236-s002.pdf]
